# An Ultrasonic System for Weed Detection in Cereal Crops

**DOI:** 10.3390/s121217343

**Published:** 2012-12-13

**Authors:** Dionisio Andújar, Martin Weis, Roland Gerhards

**Affiliations:** Department of Weed Science, Institute for Phytomedicine, University of Hohenheim, Otto-Sander-Straße 5, 70599 Stuttgart, Germany

**Keywords:** ultrasonic distance sensor, weed detection, image processing

## Abstract

Site-specific weed management requires sensing of the actual weed infestation levels in agricultural fields to adapt the management accordingly. However, sophisticated sensor systems are not yet in wider practical use, since they are not easily available for the farmers and their handling as well as the management practice requires additional efforts. A new sensor-based weed detection method is presented in this paper and its applicability to cereal crops is evaluated. An ultrasonic distance sensor for the determination of plant heights was used for weed detection. It was hypothesised that the weed infested zones have a higher amount of biomass than non-infested areas and that this can be determined by plant height measurements. Ultrasonic distance measurements were taken in a winter wheat field infested by grass weeds and broad-leaved weeds. A total of 80 and 40 circular-shaped samples of different weed densities and compositions were assessed at two different dates. The sensor was pointed directly to the ground for height determination. In the following, weeds were counted and then removed from the sample locations. Grass weeds and broad-leaved weeds were separately removed. Differences between weed infested and weed-free measurements were determined. Dry-matter of weeds and crop was assessed and evaluated together with the sensor measurements. RGB images were taken prior and after weed removal to determine the coverage percentages of weeds and crop per sampling point. Image processing steps included EGI (excess green index) computation and thresholding to separate plants and background. The relationship between ultrasonic readings and the corresponding coverage of the crop and weeds were assessed using multiple regression analysis. Results revealed a height difference between infested and non-infested sample locations. Density and biomass of weeds present in the sample influenced the ultrasonic readings. The possibilities of weed group discrimination were assessed by discriminant analysis. The ultrasonic readings permitted the separation between weed infested zones and non-infested areas with up to 92.8% of success. This system will potentially reduce the cost of weed detection and offers an opportunity to its use in non-selective methods for weed control.

## 1. Introduction

Managing patchy weed distributions is the challenge targeted by site-specific weed management. Site-specific weed control requires knowledge about spatially varying weed distribution within the field. Manual and automated weed mapping technologies have been widely explored by sampling before management operations take place (offline approach). However, they are generally expensive and not feasible for larger areas, since the weed management needs a fast reaction for weed control decisions during a single management operation (online approach). Offline methods can be based on human observation or sensing devices. Weed mapping approaches with visual assessments by humans have proven their applicability for management decisions since they are an easy tool [1]. The human eye can scan larger areas than most ground-based detectors. However, these approaches rely heavily on human perception and have various other limitations: only presence/absence or limited levels (zero/low/high) of infestation are estimated and experienced observers are required for the sampling [2]. Mapping based on sensor technology is generally accurate enough nowadays for patch spraying [3]. Online applications still have issues, since the sensor and spatial information need to be rapidly processed. The measurement and application should be possible on-the-go, not in two separate steps for mapping and application. A good weed control management often has only a narrow time window for the application. Although image recognition procedures have proven their possibilities, they still need some improvements to become commercial. Image processing for weed species recognition needs long computing times and they are not available for on-line approaches yet [4]. Although the search for automation of site-specific weed management has inspired many research developments, the sensing devices for weed detection are still a limiting factor for practical applications [5]. Since most of studies have used machine vision techniques to detect and identify plant species based on their shape, colour and texture features [6], not too many efforts have been put into the development of other sensing systems. Machine vision techniques are mainly based on digital cameras, which can be supported in a platform scanning the field with a high resolution. With a ground resolution of pixels below 1 mm^2^ the basis for species recognition is laid [7]. However, the use of these technologies for on-line applications has to be improved with faster classification algorithms and more powerful computational hardware. Concerns are still the associated costs and the relatively complex computations. The lack of commercially available systems is a major problem. The use of available optical sensors such as optoelectronic devices [8,9] have proven its possibilities. Although these sensors are not able to differentiate weeds from crops, this is not a major problem under certain conditions: in the case of row crops, all plants in the inter-row area can clearly be identified as weeds. The same applies to weed infestations occurring before crop emergence. However, the economic costs for these sensors are still high. Previous work identified plant height and biomass as important parameters for weed infestation estimation. These parameters can be estimated using ultrasonic sensors [10]. Ultrasonic sensors provide a distance measurement based on sound waves with frequencies above human hearings range. The measured travel time of an ultrasonic pulse from the emitter to the object reflecting the pulse back to the sensor is proportional to the distance. Such sensors have been available for some decades now. In the agricultural domain, they are mostly used for detection and geometric characterization of fruit tree canopies [11]. They provide an average canopy characterisation related to the leaf area index (LAI) [12,13]. Some systems were developed using ultrasonic sensors for electronic detection and real time spraying [14]. This detection procedure in combination with sprayers was used to detect the presence of trees and spray them by opening the nozzles [15,16]. Scotford and Miller (2004) [17] explored the possibilities of these devices for the estimation of tiller density and LAI. They conclude that ultrasonic sensors could be used to determine the optimum level of inputs such as fertilisers, fungicides and growth regulators at a given stage of crop development and to account for variations within a field. In addition, Fricke *et al*. [18] showed a positive forage mass-height relationship on legume-grass mixtures using ultrasonic sensors. Also, the abundance of weeds resulted in increased ultrasonic sward heights for the same levels of forage mass. Commercial products can be found for height regulation of spray booms (Dammann, Buxtehude, Germany) and growth regulator applications (Agri Con GmbH, Jahna, Germany). On the other hand, a good estimation of plant density can be calculated by the energy in the reflected wave, which is closely related to the volumetric density of the plants. Following this procedure, Swain *et al*. (2009) [19] used these sensors to discriminate weedy and bare spots in wild blueberry fields in an on-line prototype for variable-rate spraying for weed control when weeds were taller than the crop. Andújar *et al*. (2011) [20] showed the potential of this system for weed discrimination of broad-leaved weeds and grasses based on the correlation of weed biomass and ultrasonic readings in maize crops. Following the previous studies, it was hypothesised that the weed infested zones have a higher amount of biomass than non-infested areas and that this can be determined by the plant height measurements. Consequently, the possibilities of this methodology will allow the discrimination of weed infested areas, since the crop density remain homogeneous and weed patches are irregularly distributed within the field, which increases the plant density and LAI within infested areas.

## 2. Materials and Methods

### 2.1. Measurement Principle

**Figure 1.**
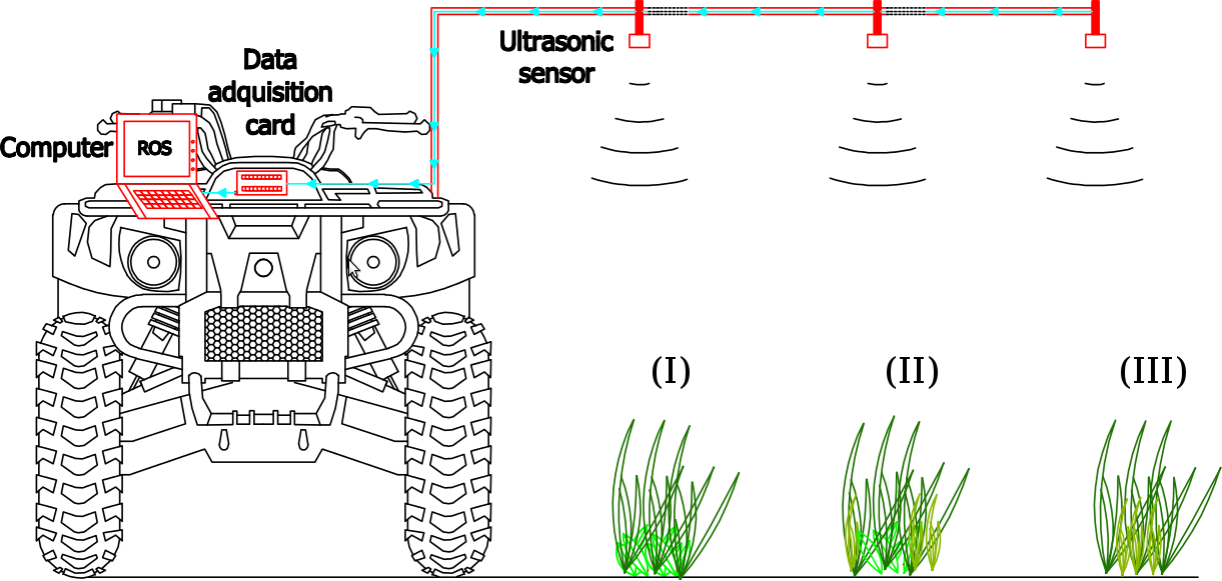
Schematic example for the ground-based ultrasonic system with the components for weed detection, working in three situations of weed–crop compositions: (**I**) crops and broad-leaved weeds; (**II**) crop and mixture of grasses and broad-leaved weeds; and (**III**) crop and grasses. Samples were taken while the vehicle was stopped.

Ultrasonic devices are based on the measurement of reflected sound waves. The estimation of the distance is based on the physical principle of time of flight, producing a short bust of sound in a unique direction. The wave returns to the receiver after impacting an object. The device measures the travel time of the acoustic signal and transforms it into a voltage signal. The output voltage can be converted into a distance. All readings were taken with a Pepperl+Fuchs (Mannheim, Germany) ultrasonic sensor (UC2000-30GM-IUR2-V15), pointing straight downward to the ground. This sensor is able to work in outdoor conditions since it has an internal temperature correction to compensate for the effect of temperature fluctuation on the electronics, a suitable degree of physical protection and a high resistance to vibrations. The ultrasonic sensor measured the distance to the crop and weed mixture covering the ground. The transducer ultrasound frequency is approximately 180 kHz with a sensor resolution of 0.48 mm when working in full evaluation range. The divergence angle was configured to result in a 0.20 m diameter footprint when placed at a height of 0.80 m (Figure 1). The response delay was used according to factory settings with a value of 195 ms. The sensing range was configured from 350 to 1,000 mm, which means that echoes generated out of this range will be signalled as erroneus measurements by the device (maximum voltage output value). A successful measurement needs a minimum surface to identify an object in the measument range.

The measurement accuracy of the device was assessed in a calibration step, where measurements over the whole range were taken and compared with the distances measured with a tape measure. The calibration equation with an R^2^ of 0.99 was established for height determination, converting the voltage signal *v* [0–10 V] into a distance *d* [cm]: *d* = 7.0275*v* + 29.658.

The sensor was connected to a 12 V battery power supply. The output was measured with a data acquisition (DAQ) module Labjack U12 (LabJack corporation, Lakewood, CO, USA) connected via USB connector to a laptop. A software was developed for ROS (Robot Operating System [21]) to acquire time stamped raw sensor (voltage) outputs and distances with the calibration equation. The heights of the crop and weeds were estimated by subtracting the actual estimated distance from the reference distance (0.80 m). This distance was acquired during an initial system calibration step. In a weed free area distance readings during 10 s were averaged to measure the height of the sensor over ground. This distance was stored as reference distance for the working conditions. The vehicle was stopped at the sample locations, and samples were taken from the continuous measurements, labelled with the infestation type and stored for the analysis.

### 2.2. Experimental Site and Measurement Procedure

Field experiments were conducted in the south west of Germany at Ihinger Hof Research Station (Renningen, Germany) of the University of Hohenheim during 2012 in a 3.5 ha winter wheat field. The experimental station Ihinger Hof (48°7″N, 8°9″E; altitude 450 m) is characterized by a mean annual precipitation slightly higher than 700 mm and a mean temperature of 9 °C. The soil of the experimental field was loam. Winter wheat was sown with 17 cm of row spacing and fertilizers were applied at planting time. A pre-emergence herbicide treatment with glyphosate was applied before sowing. Readings were taken at two dates, the first assessment was carried out on 26 and 27 March, the second on 9 April in order to cover different weed and crop stages. Weeds were assessed when the crop growth was at the stages 11 to 13 and 15 to 17 of the BBCH scale [22]. The field was mainly infested with *Echinocloa crus-galli* (L.) P. Beauv., *Lamium purpureum* L., *Galium aparine* L., and *Veronica persica* Poir. Weed growth stages ranged from BBCH 9 to BBCH 15–16 on the first sampling date and BBCH 9 to BBCH 23–24 on the second date. A total of 80 sampling points were recorded on the first date and 40 on the second date. The locations were chosen to reflect different weed compositions of grass and broad-leaved weeds as well as mixtures of both, looking for an equitable distribution of pure samples of the present species and different mixtures compositions. At these locations the sensor was pointed towards the centre of the sampled area for height determination. Immediately after taking ultrasonic readings, weed and crop densities were counted manually and the heights of the weed groups (grasses, broad-leaved weeds) and crop were determined using a metric rule.

Additionally, an RGB (red, green, blue) image was taken for weed and crop coverage determination. The images were acquired pointing the camera downwards from 1 m height. A Nikon digital camera EOS 5D (Canon Inc, Tokyo, Japan) with 6.1 megapixel DX Format CCD image sensor equipped with an 18–70 mm lens was used to capture the digital images. Then, all weeds present in a 0.20 m diameter circle, coinciding with the sensor footprint, were separately hand-harvested by groups without disturbing the ground, and taken to the laboratory for biomass determination (dry weight). The measurements were repeated after weeding: the distance was measured and a second RGB image was taken for the evaluation of the crop coverage. Finally, the crop plants were also harvested for dry weight determination.

### 2.3. Region of Interest Recognition and Image Processing

**Figure 2.**
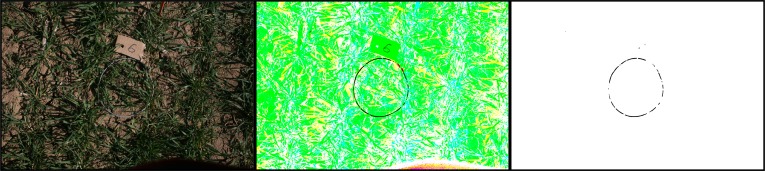
Image processing chain for frame segmentation based on colour. RGB input image (left); HSV thresholded channels combined (black: foreground, coloured/white: background), median filtered HSV colour segmentation (right).

An image sample as taken in the field is illustrated in Figure 2 (left). The area of measurement was defined using a grey circular frame, which was visible in the images. To determine the region of interest (ROI) inside the ring, an image processing chain was developed. The first step was to segment the pixels belonging to the frame based on its colour. A transformation from the RGB to the HSV (hue, saturation, value) colour space was the first step, then each of the channels was thresholded separately with pre-defined intervals (H: [116–255], S: [0–128], V: [31–255], in 8 bit channels with a value range of [0–255]). This led to three binary images with pixels falling into the selected HSV value ranges marked black (value 0, denoting object), and pixels off the range marked white (value 255, denoting background). These three binary images were combined with a binary AND operator to a single binary image (Figure 2 right). Figure 2 (centre) shows a colour combination of the three thresholded channels: the black parts denote the object pixels, any other colour is background, its colour being out of the selected range in at least one thresholded HSV channel. Small noise objects remained, these were to a large extent filtered out with a median operator of size 10, since the frame appeared as linear structure with a width of about 10–20 pixels in the image. Small regions (having a small diameter), were filtered out with this approach (Figure 2 right).

From this segmentation the region of interest defined by the inner area of the frame could be selected in the next step. Since the frame can only be segmented partially due to plant overlaps and illumination changes (shadows), the circularity of it was chosen to be a robust feature. To find a circle that fits the segmented pixels of the frame, a circular Hough transform was applied to the binary image ([23] Section 5.4.3). The Hough transform is known to be robust against noise and missing object parts (here: parts of the ring). The circular Hough transform marks all possible locations of a circle centre for a foreground pixel position in a new image, which is a representation of the Hough space, also known as the accumulator space. The Hough space was defined on a grid of the same size as the image. The circle centre candidates are arranged in a circle around the foreground (frame) pixel positions according to the circle formula (*x* − *x*_0_)^2^ + (*y* − *y*_0_)^2^ = *r*^2^. *x*_0_*, y*_0_ is the foreground pixel position and *x, y* are the possible locations of centres for a circle of radius *r*.

**Figure 3.**
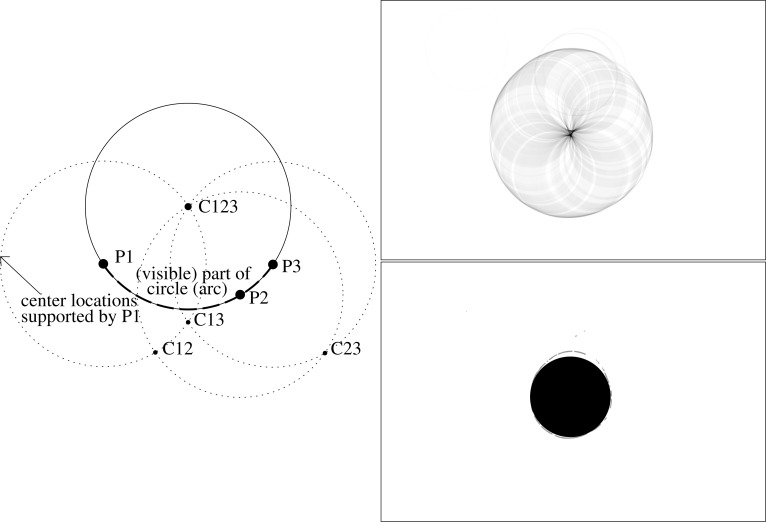
Circular Hough transform (left): the solid circle’s centre can be estimated from the partially visible (dashed) arc. For the three points P1, P2, P3 on the arc the possible centre locations can be constructed with fixed radius (dotted circles), and the intersection supported by most of the points (C123) be selected as most probable centre location. Top right: Hough space with the possible centre locations, derived from segmentation in Figure 2, bottom right: estimated circular ROI (black) with segmentation (grey).

**Figure 4.**
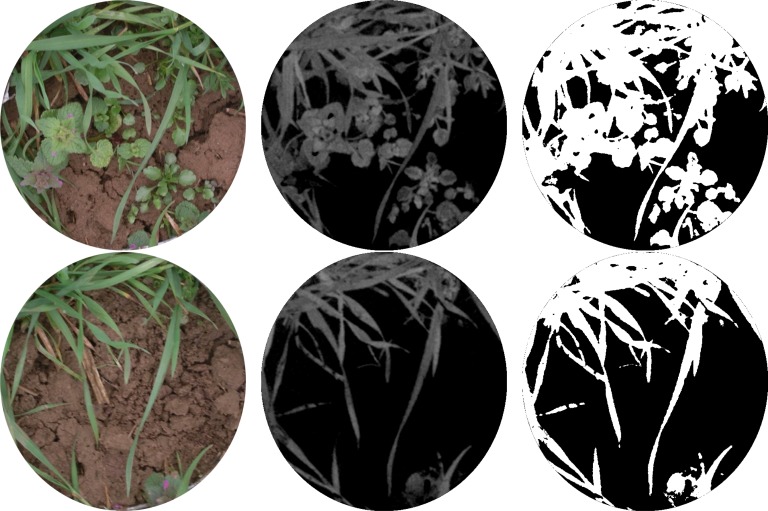
Process of image analysis before (top) and after weed harvesting (bottom). RGB images (left); ExG (Excess green) images (centre) and thresholded images (right) for coverage measurement.

In the Hough space the value in these locations (circle) around a foreground pixel is increased by one. This process is repeated for each foreground pixel. If multiple pixels are in the radius distance from a circle centre, then they have a common centre location, and the count value of that location will be increased to the number of supporting pixels. Figure 3 (left) illustrates this process for three foreground pixels P1, P2 and P3, which are all located on a circle (circle with solid line). Construction of the possible centre locations (dotted circles) leads to intersections, which are possible centre locations: C12, C23, C13 are supported by two points, C123 by all three points and therefore the latter is the most probable centre location. In the discrete Hough space (Figure 3 top right), each pixel value can be interpreted as a probability value, and the pixel with the maximum value can be selected as most probable centre location. The ROI can be constructed as circle with the radius parameter of the Hough transform. The radius of the ROI was reduced by 10 pixels to avoid parts of the frame being within the ROI (Figure 3 bottom right). This region of interest was then used to mask the RGB image (Figure 4) and define the area for the further processing. It was defined in the alpha channel of the image. The alpha channel of an image is an optional, additional channel besides RGB and contains the opaqueness (resp. transparency) for each pixel. The background was set transparent and the region of measurement to opaque, as it can be seen in Figure 4 (the part out of the circular area is transparent).

The coverage was determined before and after removal of weeds and assessed with a second image processing approach. The first step of the ROI image processing was the calculation of the ExG (excess green index, ExG = 2G-R-B [24]), which leads to a grey level image with green objects (plants) appearing bright, in contrast to objects with a different colour, which appear dark. A threshold of 8% (corresponding to grey level of 20 = 255 × 0.08) was applied to the ExG, separating the objects pixel-wise into foreground (plants) and background in a binary image (Figure 4). From the binary, image the number of foreground pixels *p_f_* and background pixels *p_b_* were counted. Only pixels within the previously defined ROI were taken into consideration. The ratio *C* = *p_f_/*(*p_b_* + *p_f_*) is then the percentage of plant coverage.

### 2.4. Statistical Analysis

Correlation analysis was conducted to provide an initial examination of bivariate relationships among the variables. Multiple linear regression analysis was then used to explore the relationship between experimental parameters that explained the differences in ultrasonic heights for the crop-weed composition in the measured combinations. A set of twelve explanatory variables, including two categorical variables, was analysed. The two categorical variables were: weed presence with two levels, and weed infestation with four levels: non-infested, infested by grasses, infested by broad-leaved weeds and infested by mixtures. The ten continuous variables were: weed coverage, grasses height, broad-leaved weed height, crop height, grasses density, broad-leaved weed density, crop density, grasses biomass, broad-leaved weed biomass and crop biomass. Regression conditions for the variables were previously tested for linearity and zero mean error, and that the variance of the error is constant across observations (homoscedasticity). The predictors were linearly independent and the errors were uncorrelated and normally distributed. A canonical discriminant analysis (CDA) was used to classify and predict the weed compositions. This methodology is a pattern recognition approach to find an optimal linear combination of input features for the separation into groups based on the dependent variable. This method expresses the dependent variable as functions of linear combinations of the input features [25]. In this study, two cases were distinguished: (I) the capabilities of the method to separate infested areas from non-infested; and (II) the possibilities of discrimination of four types of infestation levels: (a) non-infested; (b) infested by grasses; (c) infested by broad-leaved weeds and (d) infested by both weed types. The analyses were independently performed for both sampling dates using the SPSS statistical software (v. 20, IBM Corporation).

## 3. Results and Discussion

Different compositions and growth stages of weed-crop mixtures were evaluated. Wheat always showed a higher actual height than grasses and broad-leaved weeds, the latter being shorter than grasses (Table 1). The measurements led to different results depending on the assessment date. For the first date high correlations between the measured height and the variables (actual height, biomass and density) were observed while in the second date the analysis did not show a good agreement. On the first sampling date, weeds and crops were clearly distinguishable whilst in the second assessment the crop was overlapping and covering the weeds, since the latter were smaller in terms of height.

### 3.1. Correlation Analysis

**Figure 5.**
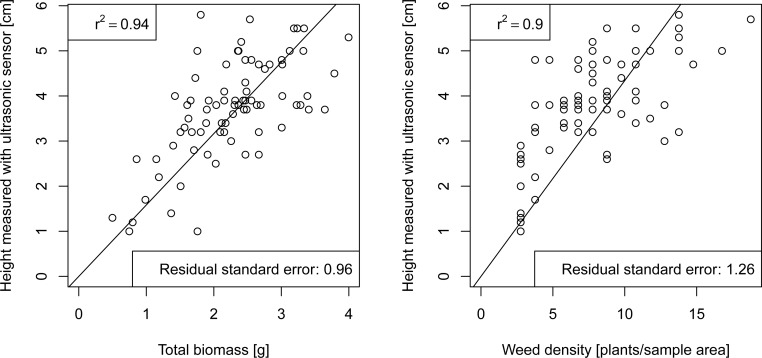
Simple linear regression plots for measured ultrasonic sensor height to weed density and total biomass (crop and weed).

High correlations between ultrasonic readings and the analysed variables for the first date were observed, these were significant on a level of *α* = 1%. Ultrasonic readings were well correlated with weed coverage reaching a Pearsons R^2^ of 0.40. For weed density and weed biomass measurements these values were higher with an R^2^ of 0.94 and 0.90 respectively. Figure 5 shows the simple regression for weed density and weed biomass with ultrasonic heights. The r^2^ denotes the correlation coefficent of the simple regression with fixed intercept (computed with the lm funciton of R statistics software). Individually, ultrasonic readings showed the highest correlations with the measured parameters of the grasses, with R^2^= 0.62 for height, R^2^= 0.67 for density and R^2^= 0.62 for biomass measurements. On the second sampling date no significant correlations were found for any of the measured parameters.

### 3.2. Multiple Regression Analysis

The comparison of the input variables indicated good relationships to the height data obtained with the ultrasonic device, with an adjusted R^2^ of 0.67 for the multiple regression model (first date). The actual height, density and biomass parameters of grass weeds had an influence in the model with a p-value >95% for the first two paramenters and a p-value >90% (Table 2). In the case of broad-leaved weeds, density was excluded from the model and actual height and biomass were significant on a level of 95% and 90%, respectively. The influence of the crop on the regression was not significant due to its constant height and biomass levels.

For the second date no significant result could be obtained. Therefore, weed detection is feasible only in early development stages of cereal crops with ultrasonic sensors.

### 3.3. Canonical Discriminant Analysis Results

The CDA was conducted for the two categorical variables. For the first sampling date, significant differences between infested and non-infested spots in the first sampling period were observed. Additionally, significant differences were found for separating infestation types of the four situations: non-infested, infested by grasses, infested by broad leaves and infested with both weeds. With a CDA satisfying results for the binary classification of the samples were achieved (Table 3). The situations were grouped into two classes (infested, non-infested). With these two groups the infested areas were correctly classified in 92.8% of the cases. Misclassified samples could be due to a later growth stage of the crop covering weeds. All non-infested cases were properly classified into the non-infested group. However, when weeds were classified into four groups, the results led to poorer results than for the binary classification, as shown in the confusion matrix in Table 4.

On the second sampling date, the use of CDA, as method for group classification, was not possible due to insignificant differences between groups. Weeds shorter than the crop were missed in cases, where the crop almost completely covered the weeds. No significant differences between groups in any of the situations were observed.

### 3.4. Comparison with other Weed Detection Principles

A previous study in maize showed the possibilities of the use of ultrasonic sensors for weed detection in the inter-row area [9]. In the case of maize, the crop did not interfere the sensor output, since the ultrasonic readings were taken in the crop-free inter-row area and the advanced growth stage of weeds slightly improved the classification results. In later growth stages the differences between weed groups were more prominent. For cereal crops the situation is different, since no crop-free areas can be measured. Due to the faster crop growth the crops cover the weeds earlier. Problems with detection occur towards later growth stages.

The effect of overlapping was dependent on the crop size, which was different for the two measurement dates. Crop plants were in both cases taller than the weeds and covered them, but the measurement principle of ultrasonic devices gives an explanation for the observed differences. The sensor generates a wave that is reflected as soon as it finds a minimum surface to create a reliable echo [26]. Since the ultrasonic measurement principle is dependent on the angle of the surface the signal hits, the minimum surface is dependent not only on the total leaf area, but also on the direction of the leaves. Plant species determination from ultrasonic signals has been explored by Harper and McKerrow (1999) [27]. In early stages, even when the crop is taller than weeds, the weeds can be measured, because the coverage by the crop is not reaching the minimum surface for echo generation. However, when the crop is in a later growth stage, the leaf coverage of the crop is large enough to reflect the ultrasonic wave and initiate an object measurement. Weeds, which were shorter than the crop, therefore did not influence the reflected echo in the second sampling. The values obtained from the second date illustrate the necessity of an early detection. A shorter, earlier time window to carry out the measurements has to be chosen, and this period of time coincides with the time window for weed treatments. Comparably, machine-vision techniques, which have shown its possibilities for ground detection of weed infestations [28], need to sample in a relatively small time window for successful weed identification. However, some advantages arise from the use of ultrasonic sensors.

The overlapping effect was reported to be a problem also for image based weed recognition approaches. In the case of image analysis, overlapping between weeds and crop make the classification more difficult [29–31], which represents a serious problem for the use of this methodology. In the case of ultrasonic measurements this effect is more dependent on the leaf size of the overlapping leaves, whereas in image processing already small leaves can lead to combined objects.

Even though ultrasonic measurements are unable to identify weed infestation separated by species, they can be used for the application of herbicides (or mixtures) with a broad spectrum. This approach was used for the development of a prototype automated variable rate sprayer for on-line application of agrochemicals in wild blueberries [32]. The researchers also concluded that the use of the ultrasonic measurements are accurate enough for patch spraying and suggested the possibility of detection at early stages, similarly to the situation found in cereal crops. Indeed, one of the major advantages of its use would be the possibility of its use for real time operation. Patch spraying can be controlled directly according to the measurements, without the need for an additional previous sampling and mapping step. The ultrasonic output is easy to process and the high number of readings per second lead to a high spatial sampling resolution in the direction of travel. Another advantage is the relatively low costs of these sensors that allow using multiple sensors in parallel. Thus, the whole field can be scanned with a high spatial resolution, which is necessary for a detailed application decision for patch spraying.

Compared with optoelectronic sensors, the obtained results of this study reveal a potential of this technique to additionally assess weed density, not only coverage [33]. Since the measurement principle is non-optical, ultrasonic sensors could be combined with optoelectronic sensors (sensor fusion, as proposed by Adamchuk *et al.*[34]) to obtain more reliable estimations especially for low infestations. The application window for on-field operations could be extended this way. No interferences between the two types of sensors are expected, since the measurement is based on different principles (light, sound). Additionally, the ultrasonic sensor principle is not affected by ambient light conditions, thus it can be applied during night hours without the need for advanced illumination. The costs for the sensors are lower than for optoelectronic systems, providing a budget solution for real-time applications to the end-users.

## 4. Conclusions

A complete weed detection sensor system needs to integrate the sensors, the results of this study and processing units to generate control information for the application equipment. Additionally, an adaptable decision component should be part of such a system, taking into account a priori knowledge about the infestation types and the possibilities of the application technology.

The presented sensing methodology can determine weed infestations in cereals as long as the cereal crop is not too developed. The system validation using actual parameter showed that weed presence was correctly predicted in more than 92% of the cases. The sensors are relatively cheap and easy to integrate into real-time applications, which would allow a reduction in detection and patch spraying. However, small differences in readings between infested and non-infested areas require further experiments to be conducted before market-readiness can be achieved.

## Figures and Tables

**Table 1.** Descriptive statistics for the variables of the study (in cm). Correlations were computed for ultrasonic heights and N is the number of cases.

	**Correlations**	**N**	**Minimum**	**Maximum**	**Mean**	**Std. Deviation**
Ultrasonic height	1.00	80	1.0	5.8	3.7	1.0
weed coverage	0.40	68	.05	40.6	16.7	10.3
grasses height	0.62	59	3.0	5.0	3.6	0.6
broad-leved weed height	0.15	30	1.0	6.0	2.0	1.1
crop height	0.32	80	5.5	8.0	6.6	0.5
grass weed density	0.67	59	1.0	14.0	4.8	2.9
broad-leaved weed density	0.05	30	1.0	12.0	3.5	2.9
crop density	0.19	80	6.0	13.0	9.7	1.7
grasses biomass	0.62	57	0.0	1.9	0.7	0.4
broad-leaved biomass	0.07	28	0.0	1.1	0.3	0.3
crop biomass	0.23	80	0.5	2.8	1.6	0.5

**Table 2.** Multiple regression analysis between ultrasonic readings and measured coverage, density and biomass for each measured variable (n.s.: not significant).

	**First sampling date**			**Second sampling date**		

	Coefficient *	**Std. error**	**P-value**	Coefficient *	**Std. error**	**P-valor**
Weed coverage	0.029	0.011	0.009			n.s
Grasses height	0.301	0.065	0.04			n.s
Broad-leaves height	0.227	0.089	0.023			n.s
Crop height			n.s			n.s
Grasses density	0.392	0.041	0.03			n.s
Broad-leaves density			n.s			n.s
Crop density	0.225	0.042	0.001			n.s
Grasses biomass	0.174	0.254	0.09			n.s
Broad-leaves biomass	0.177	0.456	0.085			n.s
Crop biomass			n.s			n.s

* Standardised regression coefficients; R^2^ = 0.667 for the model between the dependent variable (ultrasonic reading) and all significant independent variables as listed in the table.

**Table 3.** Canonical discriminant classification showing the percentage of correct group classification in a binary classification (first sampling date).

	**Predicted**	
	infested	non-infested
Infested	92.8	7.2
Non-infested	0	100

**Table 4.** Confusion matrix of the canonical discriminant classification showing the percentage of correct group classifications with four groups (first sampling date).

	**Predicted**			
	non-infested	grass weed	broad-leaved weed	both weed types
non-infested	90.9	0.0	9.1	0.0
grass weed	2.6	41.0	28.2	28.2
broad-leaved weed	30.0	40.0	30.0	0.0
both weed types	0.0	30.0	35.0	35.0
